# Extrahepatic metabolism of ibrutinib

**DOI:** 10.1007/s10637-020-00970-x

**Published:** 2020-07-04

**Authors:** Johannes J. M. Rood, Amer Jamalpoor, Stephanie van Hoppe, Matthijs J. van Haren, Roeland E. Wasmann, Manoe J. Janssen, Alfred H. Schinkel, Rosalinde Masereeuw, Jos H. Beijnen, Rolf W. Sparidans

**Affiliations:** 1 Division of Pharmacoepidemiology & Clinical Pharmacology, Faculty of Science, Department of Pharmaceutical Sciences, Utrecht University, Universiteitsweg 99, 3584 CG Utrecht, The Netherlands; 2 Present Address: Benu apotheek Hoorn, Pakhuisstraat 80, 1621 GL Hoorn, The Netherlands; 3 Present Address: Division of Pharmacology, Faculty of Science, Department of Pharmaceutical Sciences, Utrecht University, Universiteitsweg 99, 3584 CG Utrecht, The Netherlands; 4 Division of Pharmacology, The Netherlands Cancer Institute, Plesmanlaan 121, 1066 CX Amsterdam, The Netherlands; 5 Present Address: Charles River Laboratories, Darwinweg 24, 2333 CR Leiden, The Netherlands; 6 Division of Chemical Biology & Drug Development, Faculty of Science, Department of Pharmaceutical Sciences, Utrecht University, Universiteitsweg 99, 3584 CG Utrecht, The Netherlands; 7 Present Address: Institute of Biology, Biological Chemistry Group, Leiden University, Sylviusweg 72, 2333 BE Leiden, The Netherlands; 8 Department of Pharmacy, Radboud University Medical Centre, Geert Grooteplein Zuid 10, 6525 GA Nijmegen, The Netherlands; 9 Present Address: Division of Clinical Pharmacology, Department of Medicine, University of Cape Town, Observatory, Cape Town, 7925 South Africa; 10 Department of Clinical Pharmacology, The Netherlands Cancer Institute, Plesmanlaan 121, 1066 CX Amsterdam, The Netherlands

**Keywords:** Ibrutinib, Metabolism, Glutathione cycle, Pharmacokinetics, Bioactivation, LC-MS/MS

## Abstract

Ibrutinib is a first-in-class Bruton’s kinase inhibitor used in the treatment of multiple lymphomas. In addition to CYP3A4-mediated metabolism, glutathione conjugation can be observed. Subsequently, metabolism of the conjugates and finally their excretion in feces and urine occurs. These metabolites, however, can reach substantial concentrations in human subjects, especially when CYP3A4 is inhibited. Ibrutinib has unexplained nephrotoxicity and high metabolite concentrations are also found in kidneys of Cyp3a knockout mice. Here, a mechanism is proposed where the intermediate cysteine metabolite is bioactivated. The metabolism of ibrutinib through this glutathione cycle was confirmed in cultured human renal proximal tubule cells. Ibrutinib-mediated toxicity was enhanced in-vitro by inhibitors of breast cancer resistance protein (BCRP), P-glycoprotein (P-gp) and multidrug resistance protein (MRP). This was a result of accumulating cysteine metabolite levels due to efflux inhibition. Finally, through inhibition of downstream metabolism, it was shown now that direct conjugation was responsible for cysteine metabolite toxicity.

**Electronic supplementary material:**

The online version of this article (10.1007/s10637-020-00970-x) contains supplementary material, which is available to authorized users.

## Introduction

The first-in-class covalent Bruton’s Tyrosine Kinase (BTK) inhibitor ibrutinib can inactivate the NFκB pathway. It is registered for mantle cell lymphoma (MCL), chronic lymphocytic leukemia (CLL) [1], Waldenström’s macroglobulinemia [2], marginal zone lymphoma, and chronic graft versus host disease [3] and has proven to be a milestone therapy in CLL. Ibrutinib is a targeted covalent inhibitor, so after an initial non-covalent interaction with the target, it binds irreversibly to a free cysteine near the target site [4, 5]. The primary route of ibrutinib metabolism is through Cytochrome-P450 (CYP). Ibrutinib is mainly metabolized to dihydrodiol-ibrutinib (DHI) by CYP3A4 and to a lesser extent by CYP2D6 [6]. While CYP-mediated metabolism of ibrutinib has been well characterized, the knowledge on its extrahepatic clearance is limited. This route may gain importance when oxidative CYP metabolism is impaired by, for example, concomitant medication or genetic defects. Glutathione (GSH) and glutathione *S*-transferase (GST) play an important role in detoxification processes and are highly expressed in the cytosol of various cells from the liver and kidney [7]. Synthesis of GSH by the ɣ-glutamyl cycle is important for maintaining GSH homeostasis and a normal redox status. The extracellular ɣ-glutamyl transpeptidase (ɣ-GT) plays a key role by breaking down extracellular GSH and providing the rate-limiting substrate cysteine (CYS) for de novo synthesis of GSH [8]. GSH and GSH-conjugates are metabolized through ɣ-GT to cysteinyl-glycine (CGS) and CGS-conjugates, which are further metabolized to CYS(−conjugates) by dehydropeptidase I (renal membrane dipeptidase). The conjugates are eliminated after acetylation by *N*-acetyl transferase (NAT) to mercapturic acid-conjugates [8].

Shibata and Chiba [9] noted a discrepancy between predicted clearance from hepatocyte incubations and total body clearance of ibrutinib. This was attributed to extrahepatic conjugation to GSH, which was not captured in the in vitro hepatocyte system. Current in vitro studies utilizing hepatocytes or microsomal systems to evaluate CYP-mediated metabolism of compounds like ibrutinib may therefore overestimate the contribution of these enzymes to the clearance of ibrutinib in vivo. Because of this over-estimation, non-oxidative metabolism through glutathione could have a more prominent role in-vivo than was previously expected, especially when oxidative metabolism is impaired, as for instance by drug-drug interactions [10]. Accurate prediction of clinical drug-drug interactions depends on the understanding of the metabolism and disposition of the drug, including elucidation of the fraction of drug metabolized through these various pathways.

Renal toxicity of ibrutinib is reported frequently, with effects ranging from an increased plasma creatinine to lethal kidney failure [11]. In one trial of 111 MCL patients treated with ibrutinib, three patients (2.7%) developed acute renal failure [12]. The first biopsy-proven cases in literature showed acute tubular injury [11], the mechanism of this drug-related injury is still unclear. In addition, ibrutinib may induce tumor lysis syndrome [11, 13], a clinical complication that may lead to acute kidney injury and severe hyperuricemia. Thus, in this study we aimed to gain a complete picture of ibrutinib metabolism in the human body and understand the observed kidney toxicity.

The role of Ibrutinib’s metabolization pathway is important to understand for the accurate prediction of clinical drug-drug interactions and to understand its nephrotoxic properties. In addition, drug transporters like the breast cancer resistance protein (BCRP (MDR1/*ABCB1*)), P-glycoprotein (P-gp (*ABCG2*)) and multidrug resistance-associated proteins 2 and 4 (MRP2/4 (*ABCC2/4*)) can play a role in the distribution of ibrutinib and its metabolites. We therefore measured the small molecular thiol (SMT)-metabolites of ibrutinib in patient plasma samples and in vitro using human conditionally immortalized proximal tubule epithelial cells (ciPTEC) [14]. Proximal tubules are vital for the reabsorption of filtered solutes from the glomerular filtrate of the kidneys and are known to be vulnerable to cysteine conjugates through their active uptake and presence of beta-lyase [15, 16]. This enzyme can bioactivate cysteine-*S*-conjugates and may convert ibrutinib to ibrutinib-thiol (ibrutinib-SH), thereby potentially leading to nephrotoxicity [17–19]. In addition, renal cells have a high expression of GSH-metabolizing enzymes, like renal dipeptidases. These enzymes facilitate the metabolism of CGS-conjugates to CYS-conjugates. The human urine-derived ciPTEC have been well characterized and have demonstrated extensive metabolic activity [20]. Hence, next to the evaluation of extrahepatic metabolism of targeted covalent inhibitors, ciPTEC could act as a suitable model to assess renal toxicity induced by ibrutinib-thiol metabolites.

## Methods

### Chemicals

Ibrutinib (>99.9%, Mw: 440.51 g/mol) was obtained from LC Labs (Wyoming, MA, USA). All chemicals were from commercial courses and were at least of analytical grade. Sources of analytical standards and chemicals for chromatographic methods were reported previously [21].

### Synthesis and analysis of ibrutinib and its metabolites

The synthesis and analysis of the ibrutinib-SMT compounds IGSH, ICGS, and ICYS was published previously [21]. In short, the corresponding SMT’s were incubated with ibrutinib, purified and freeze-dried. For analysis, 200 μL acetonitrile containing the internal standards was added to 100 μL sample. After mixing and centrifuging, 200 μL supernatant was transferred, and thereafter the solvent was partially evaporated. Before the samples were analyzed by ultra-high-performance liquid chromatography with mass spectrometer detection [21], 100 μL of mobile phase (water:acetonitrile:formic acid, 90:10:0.1 (v:v:v)) was added.

### Ibrutinib and SMT-conjugate pharmacokinetics in human subjects

Time-concentration data of three patients were analyzed. Exact sampling times of patients A and B were unknown and therefore these samples were grouped as ‘abs’ (<2 h), ‘max’ (≥2 h and < 6 h), ‘inter’ (≥6 h and < 12 h), and ‘trough’ (≥12 h). When no time was available at all, the sample was omitted. Samples were taken for therapeutic drug monitoring purposes in standard treatment with ibrutinib. No interventions were made on behalf of this retrospective study. Pharmacokinetic (PK) data was analyzed using the PKNCA package (version 0.8.1) [22] for the statistical software R (version 3.4.2) [23] and RStudio (version 1.1.383) [24].

### Metabolism of ibrutinib in ciPTEC

CiPTEC (MTA number A16–0147, passage 35–40) were obtained from Cell4Pharma, Nijmegen, The Netherlands and developed as described previously [14]. Briefly, cells were retrieved from urine from a healthy volunteer in compliance with the guidelines of the Radboud Institutional Review Board and conditionally immortalized via transduction with the temperature-sensitive mutant of SV large T antigen (SV40T) and human telomerase reverse transcriptase (hTERT). CiPTEC were seeded in a 6-well plate and incubated at 37 °C and 5% (*v*/v) CO_2_ with serum free medium containing either 2 μM ibrutinib or 1 μM IGSH for 4 h. To study the kinetics of ibrutinib and the ISMT metabolites, cells were incubated with ibrutinib (2 μM). Medium and cell lysate samples were taken at 1, 2, 3 and 4 h. Each time point was measured in duplicate. Preincubation (t_0_) analyte concentrations in medium were also analyzed. To distinguish direct conjugation of ibrutinib to ICYS, inhibitors of the GSH-cycle were used. For ɣ-GT, acivicin (IC_99_: 50 μM [25]), for dehydropeptidase I, cilastatin (IC_50_: 100 μM [26]) and for GST, ethacrynic acid (IC_97_: 15 μM [27]), all were used at 100 μM. CiPTEC sample collection is described below.

### CiPTEC sample collection

During sample collection the samples were kept on wet ice, mainly to prevent degradation of the IGSH metabolite. After each experiment, the media were collected in 2 mL polypropylene reaction vials. The cells were washed twice with 2 mL ‘Hank’s Balanced Salt Solution’ (HBSS, Thermo Fisher, Ermelo, The Netherlands). The cells were detached by adding 300 μL Accutase™ cell detachment solution (BD, Etten-Leur, The Netherlands), and incubated at 33 °C for 5 min. Hereafter, 1 mL HBSS was added to suspend the cells and inactivate the Accutase. The cells were collected in a 1.5 mL reaction vial. To remove any residual medium, the cells were washed twice with HBSS by means of centrifuging at 250 x *g* and 4 °C during 5 min. The supernatant was hereafter discarded and 1 mL HBSS was added to resuspend the cells. Finally, the cell pellet was snap frozen in liquid nitrogen, after which the cells were sonicated three times for the duration of 10 s with 20 s cooling intervals (cycle: 1, amplitude: 80%, Hielscher UP50H, Teltow, Germany). Finally, the samples were pre-treated according to the standard analysis protocol [21].

### Real-time reaction monitoring of conjugation by LC-MS/MS

To investigate the kinetics of ibrutinib (10 μM) in the thiol pool samples, a real time LC-MS/MS experiment was performed [28]. The autosampler was set to 37 °C, 990 μL of 50 μM CYS, GSH and control (PBS) were pre-incubated in the autosampler for 10 min. Ten μL of 1 mM ibrutinib stock solution was quickly added to the autosampler vial right before the injector started to process the sample.

One μL was injected onto a BEH300 C18 column (2.1 × 50 mm, d_p_ = 3.5 μm, Waters, Milford, USA). Gradient elution was performed using 0.1% formic acid in water (A), and methanol (B). The elution program started isocratically at 70% B from 0 till 36 s, and methanol was increased linearly to 95% at 48 s, where the composition returned to 70% B till 60 s. The whole eluate was transferred to the MS, which was operated as described previously [21]**.** All reactions were measured in 3-fold. The reaction kinetics were calculated using Prism 6.0 h (GraphPad Software, La Jolla, CA, USA).

### Monitoring bioactivation metabolites of ibrutinib

To monitor the bioactivation of ICYS, the predicted selected reaction monitoring (SRM) transitions for the Ibrutinib-SH, *S*-methyl ibrutinib, and ibrutinib-*S*-glucuronide were used. The fragments were predicted based on the fragmentation-patterns of ibrutinib and the SMT-metabolites, with the signature fragment found at m/z 304.1. The predicted SRM values and the accompanying MS parameters are shown in Table 1. For ibrutinib-*S*-glucuronide, the typical neutral loss fragment of a glucuronic acid group (−176 Da) was also used.

**Table 1:** Predicted SRM-transitions for the bioactivation metabolites Ibrutinib-SH, S-methyl-ibrutinib, and ibrutinib-S-glucuronide. The entrance potential was set at 10 V for all compounds, dwell-time was 5 ms.

Compound	Q1	Q3	DP	CE	CXP
	(m/z)	(m/z)	(V)	(V)	(V)
Ibrutinib-SH^a^	475.184	304.1	171	43	12
	475.184	172.1	171	35	20
S-methyl-ibrutinib	489.199	304.1	171	43	12
	489.199	186.1	171	35	20
Ibrutinib-sulphenic acid	491.186	304.1	211	43	12
	491.186	188.1	211	35	16
	491.186	84.1	211	73	10
Ibrutinib-S-glucuronide	651.216	473.1	171	40	12
	651.216	475.2	171	35	12
	651.216	304.1	171	43	12

^a^reactive metabolite

### Transport of ibrutinib and its metabolites in ciPTEC

Transporters involved in the transport of ibrutinib and the thio-metabolites were investigated by co-incubating ibrutinib with BCRP, P-gp and MRP inhibitors, KO143, PSC833, and MK571, respectively for 4 h. Each inhibitor was used at 5 μM. The conditions tested were 2 μM ibrutinib with either MRP, MRP + BCRP, MRP + P-gp or BCRP + P-gp-inhibition, or vehicle (control) (*n* = 2 per condition). CiPTEC cells were pre-incubated for 30 min with the respective inhibitors. Unmatched two-way ANOVA was used to test for significance.

### Cell viability assay

CiPTEC were seeded in a 96-well plate as described and treated for 48 h with 2 μM ibrutinib both in presence and absence of inhibitor combinations (described above). Cytotoxicity was evaluated using a Presto-blue assay as follows: cells were incubated for 1 h with Presto-blue solution (100 μL/well) at 37 °C and absorbance read using a Jasco FP8300 fluorometer (Tokyo, Japan; excitation wavelength: 560 nm, emission wavelength: 590 nm).

### Glutathione*-S-*Transferase assay

To assess the involvement of GST, ibrutinib was incubated with either HLMs, liver cytosol, S9 fraction (all: Corning, Tewksbury, MA), or enzyme free control. IGSH formation after 1 h was used as a measure of GST-involvement. One microliter of HLMs (20 mg/mL), cytosol, S9-fraction or water was transferred to a 1.5 mL reaction vial with 1 mM GSH, 0.1 mM Na-EDTA, 50 mg/mL bovine serum albumin, and 100 mM phosphate buffer (pH 7.4). The reaction was started by addition of ibrutinib (final concentration: 500 nM). After 1 h the reaction was quenched using the standard analysis protocol [21]. Separately, the GST activity in ciPTEC lysate was assayed. Before adding ibrutinib and GSH, 25 μL lysate was pre-incubated for 2.5 h with and without 100 μM ethacrynic acid. GSH, EDTA and ibrutinib were added (final concentrations 1 mM, 0.1 mM, and 500 nM). After 4 h the reaction was quenched using the standard analysis protocol [21].

### ɣ-glutamyl transferase assays

IGSH was incubated with 50 mU/L ɣ-GT in a concentration range of 10–10,000 nM in phosphate buffer (100 mM, pH 7.4) at 37 °C. Michaelis-Menten kinetics were calculated by Eadie-Hofstee transformation. Additionally, the half-life of IGSH was tested in 1 mL human plasma at 37 °C, at 500 nM. Samples of 100 μL were taken, and the reaction was quenched using the standard analysis protocol [21].

For ɣ-GT inhibition, ciPTEC cells were pre-incubated with 100 μM acivicin for 2.5 h before adding ibrutinib (2 μM). The formation of metabolites in medium and cells was monitored after 4 h. Ɣ-GT activity in human plasma and calf serum was measured on a Beckmann Coulter AU5800 (Fullerton, CA, USA) clinical chemistry analyzer, with a limit of quantitation of 1 U/L.

### SMT-conjugate formation in mice

FVB/NRj (Janvier lab, Le Genest-Saint-Isle, France) and Cyp3a^−/−^ (FVB/NRj genetic background; RRID:IMSR_TAC:9011) mice were housed and handled as reported previously [29] and were treated with 10 mg/kg ibrutinib orally. Serial blood samples (≤50 μL) were collected in lithium heparin-containing microvettes (Sarstedt, Germany) via the tail vein at 5, 10 and 15 min (peak concentration). After 20 min, the mice were sacrificed, blood was collected by hart puncture and liver, spleen, brain and kidneys were harvested to evaluate tissue accumulation of the SMT-conjugates. The FastPrep-24™ 5G instrument (M.P. Biomedicals, Santa Ana, CA, USA) was used for 1 min to homogenize tissues in 4% (*w*/*v*) bovine albumin solution. The volumes of albumin solution used were 3 mL for a liver, 2 mL for two kidneys, and 1 ml for brain and spleen. Plasma was obtained from blood by centrifugation at 9000 x *g* for 6 min at 4 °C. These measurements were part of an in vivo experiment conducted by van Hoppe et al. [30].

## Results

### Pharmacokinetics of ibrutinib and its metabolites in human plasma

**Fig. 1:**
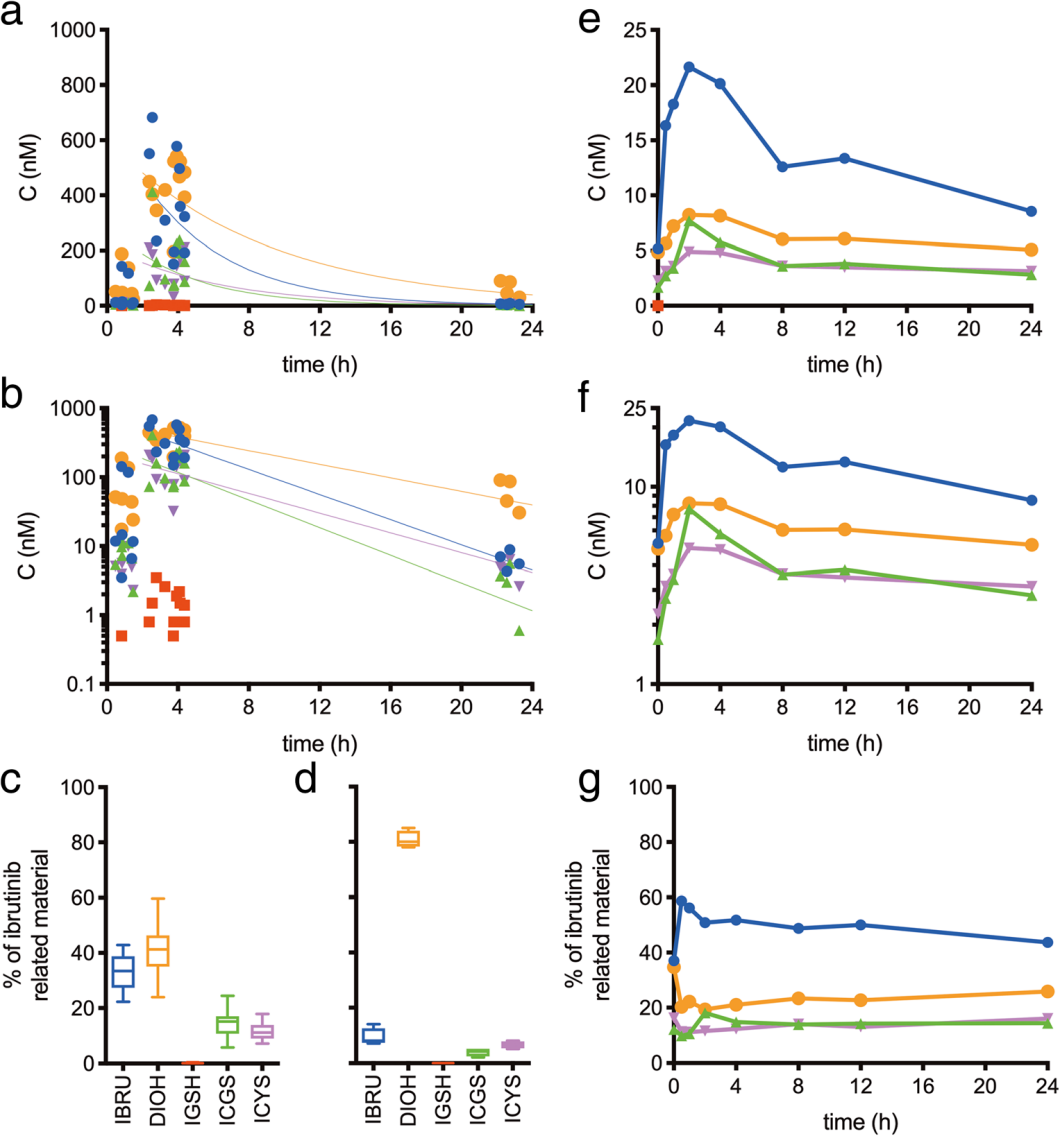
Linear (**a**) and log-transformed (**b**) concentration-time points of patients A and B receiving ibrutinib and the fraction of ibrutinib related material (relative to sum of quantified compounds) around T_max_ (**c**) and T_trough_ (**d**). Linear (**e**) and log-transformed (**f**) concentration-time curve of ibrutinib (), IGSH (), ICGS (), ICYS (), and DHI () for patient C, receiving 140 mg ibrutinib through a nasogastric tube with concomitant voriconazole, and the calculated fraction of ibrutinib related material (G, relative to sum of quantified compounds)

To evaluate the extrahepatic metabolism of ibrutinib, plasma levels of two patients, patient A (male, 73 y) and patient B (male, 75 y) treated with either 420 mg or 560 mg ibrutinib once daily were analyzed in time (Fig. 1a/b) and in relative levels (Fig. 1c/d). Mean plasma C_max_ (‘max’, *n* = 11) and C_trough_ (‘trough’, *n* = 4) levels are summarized in Table 2. Data points that were part of the absorption phase (‘abs’, *n* = 7), or of which the time was not known (*n* = 2), were not used for PK analysis for determination of the elimination half-lives, but were included in the overall plasma-levels (Supplemental Table 1).

**Table 2:** Overall, maximum, and trough levels, times of maximum concentration (mean ± SD (range)), and estimated half-lives for ibrutinib, DHI, IGSH, ICGS, and ICYS for patients A and B

	C_overall_ (nM)	C_max_ (nM)	C_trough_ (nM)	T_max_ (h)	T_1/2_ (h)
Ibrutinib	184 ± 214 (< 0.5–683)	370 ± 180 (151–683)	6.4 ± 2 (4.3–8.9)	2.6	3.3
DHI	242 ± 200 (<0.5–542)	432 ± 99.6 (195–542)	63.3 ± 30 (30.5–90.7)	3.9	6.1
IGSH	1.5 ± 0.92 (<0.5–3.5)	1.59 ± 0.91 (0.5–3.5)	<0.5	2.8	a
ICGS	81.3 ± 106 (<0.5–413)	163 ± 103 (72.5–413)	3.2 ± 2.1 (0.6–5.6)	2.6	3
ICYS	67.8 ± 78.3 (<0.5–211)	134.9 ± 63 (32.1–211)	4.7 ± 1.6 (2.6–6.4)	4.4	4.2

^a^could not be calculated

### Human ibrutinib metabolism during CYP3A4 inhibition

A third patient (patient C, male, 73 y), treated with ibrutinib for small lymphocytic lymphoma, was presented with a fungal infection with cerebral lesions. He was admitted to the ICU for ventilation and treatment with voriconazole (twice daily 340 mg intravenously in 1 h), a potent CYP3A4 inhibitor. Due to the voriconazole, a severe ileus, and renal insufficiency, extensive blood levels sampling was performed on day 5 of ICU admission. Plasma-samples were taken at 0 h (pre-dose), 0.5, 1, 2, 4, 8, 12, and 24 h. The patient was given a lowered dose of 140 mg ibrutinib (once daily by nasogastric tube) due to the concomitant inhibitor a complete list of comedication is added as Supplemental Table 2. The plasma concentration-time curve is shown in Fig. 1e/f and relative amounts in Fig. 1g. The area under the concentration-time curve at steady state (AUC_0-24h,ss_), half-lives, and relative exposure to that of ibrutinib and to that of the total related compounds are depicted in Table 3.

**Table 3:** AUC_0-24h,ss_, relative exposures and estimated half-lives for ibrutinib, DHI, and ibrutinib-SMT compounds in patient C

Compound	AUC_0-24h,ss_	Exposure relative to	Sum of quantified	T_1/2_
	(nmol·h/L)	ibrutinib	compounds	(h)
Ibrutinib	324.7	–	49%	18.5
DHI	149.5	46%	23%	59.0
IGSH	a	a	a	a
ICGS	94.9	29%	14%	40.1
ICYS	87.7	27%	13%	78.3

^a^could not be calculated, levels below lower limit of quantitation

### CiPTEC demonstrate active metabolism of ibrutinib

**Fig. 2:**
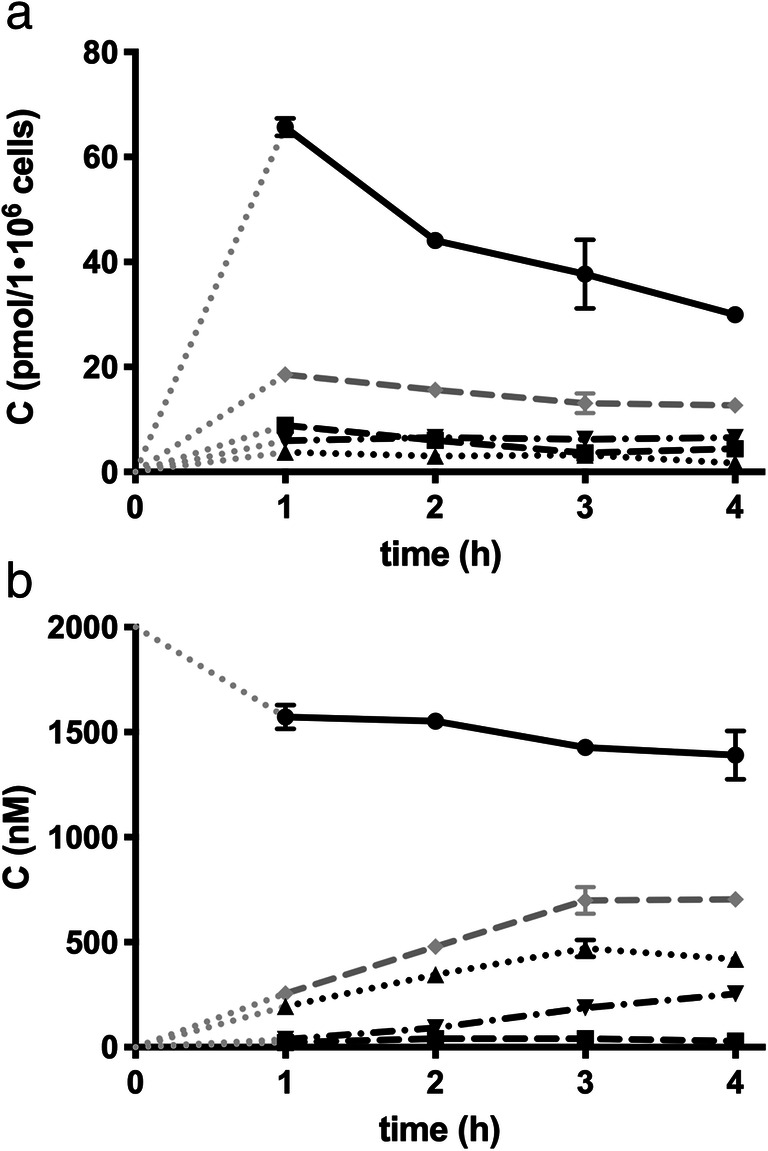
Kinetics of ibrutinib (●), IGSH (■), ICGS (▲), and ICYS (▼) in ciPTEC cells. **a**) Intracellular concentrations of ibrutinib and ISMT’s. **b**) culture medium-concentrations of ibrutinib and ISMT’s. The grey dotted lines (^. . . . . .^) represent the intrapolated data from t_0_ to t_1_, the grey dashed lines (–  –  –  –) represent the sum of al ISMT

**Fig. 3:**
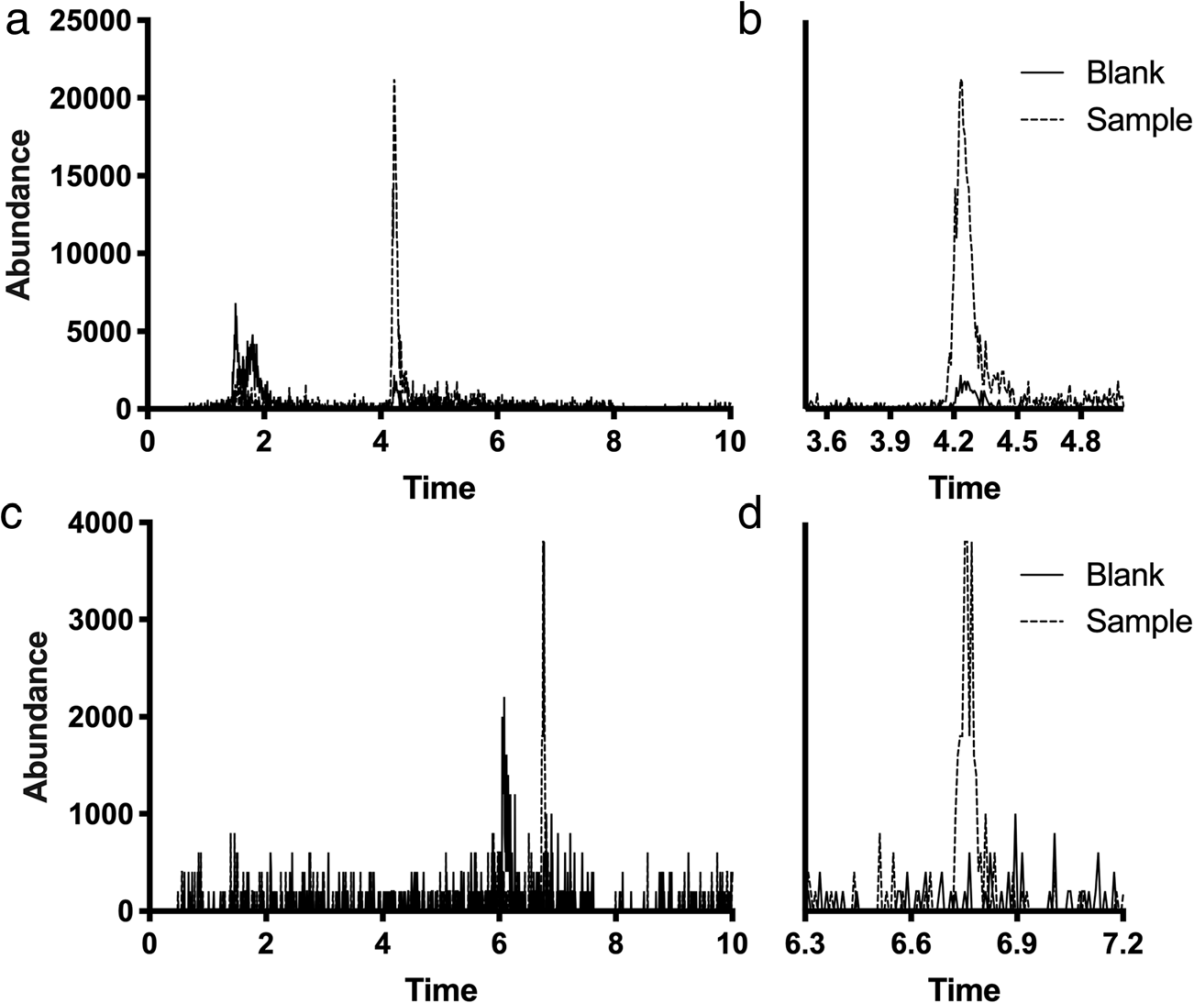
Chromatograms of **a**/**b**) ibrutinib-S-glucuronide (m/z 651.2 > 304.1) in ciPTEC cells after 4-h incubation with ibrutinib and **c**/**d**) S-methyl ibrutinib (m/z 489.2 > 304.1) in ciPTEC cells after 48 h with ibrutinib (–  –  –  –), or blank (──)

Human proximal tubule cells, ciPTEC, were used to investigate the extrahepatic metabolism of ibrutinib [14]. In these cells, ibrutinib showed a half-life of 18.8 ± 9.3 h (value±SD) and a CL_int_ of 87 pmol/h/1·10^6^ cells (Fig. 2). At 4 h, the bioactivation of ICYS was monitored by the predicted SRM transitions for ibrutinib-SH, *S*-methyl ibrutinib, ibrutinib-sulphenic acid, and ibrutinib-*S*-glucuronide (Fig. 3). The ibrutinib-*S*-glucuronide was the only secondary bioactivation-metabolite of ICYS that was clearly found in ciPTEC (Fig. 3a/b). After 48 h, ibrutinib was completely metabolized (ca. 98%), and only ICYS metabolite remained. In addition, *S*-methyl-ibrutinib could also be detected (Fig. 3c/d).

During ibrutinib incubations, IGSH was formed intracellularly and transported extracellularly where it was metabolized to ICGS and further to ICYS. IGSH and ICYS were mainly found intracellularly. Incubating with IGSH directly for 4 h revealed that there is no uptake of IGSH and the downstream metabolites ICGS and ICYS. This indicates that the ICYS metabolite might also be formed by direct conjugation with cysteine. Medium composition influences the metabolism of IGSH. IGSH was metabolized 32% faster in medium with 10% calf serum, when compared to serum-free samples. Medium that was pre-incubated with ciPTEC for 48 h (conditioned medium) was included in the experiments to investigate the possible role of secreted enzymes in the medium. The metabolism of IGSH to ICGS appeared to be 36% faster in conditioned medium. However, conditioned medium had no effect on ibrutinib metabolism itself (data not shown).

### Real-time reaction monitoring of GSH-dependent conjugation

**Fig. 4:**
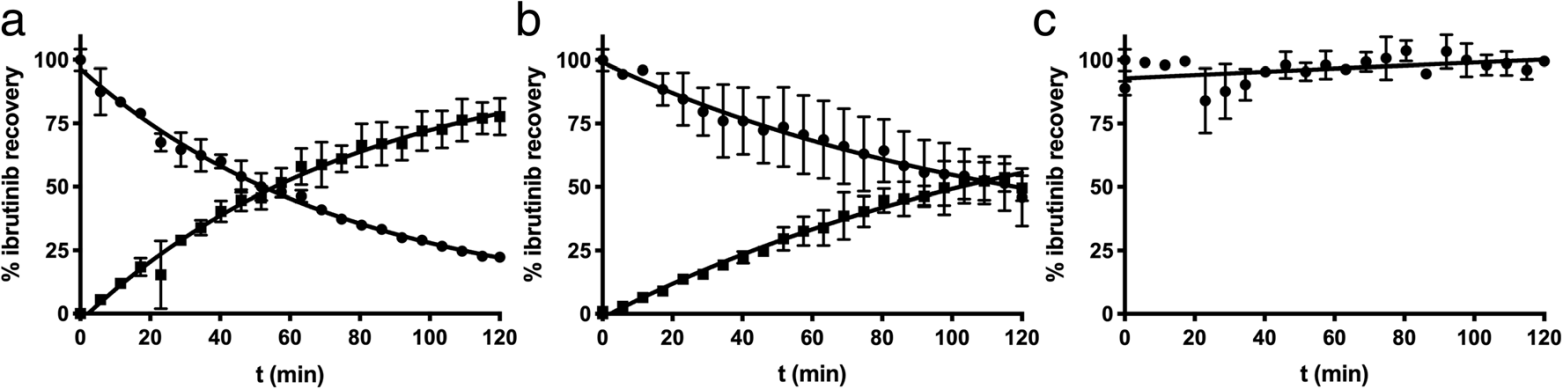
Real time reaction kinetics of 1 mM ibrutinib (●) and the appearance corresponding ISMT (■) in the presence of 10 mM GHS (**a**), CYS (**b**), or control (**c**). Each data point is represented as mean ± sd

To determine the reactivity of ibrutinib towards GSH and CYS, a real-time reaction monitoring setup was used. The ibrutinib concentration in the incubated samples decreased over time, while the ibrutinib-conjugate concentration increased compared to the unchanged control group. The reaction half-lives of ibrutinib in 50 μM CYS and GSH were 54.5 min (95%CI: 52.2–56.9 min) and 116.1 min (95%CI: 91.16–104.0 min), respectively. The decrease of ibrutinib levels inversely corresponded to the formation of IGSH or ICYS (Fig. 4).

### Involvement of glutathione-*S*-Transferase in ibrutinib metabolism

While there is a decrease in IGSH formation in human liver microsome (HLM) incubations when compared to non-enzymatic conjugation, cytosol shows considerable GSH conjugation compared to the non-enzymatic incubation. This indicates the involvement of human liver cytosol GST enzymes, although the subclass of the enzymes could not be elucidated due to the lack of specific inhibitors. Maximal reactivity with ibrutinib was found at 1000 μM GSH. Due to an incomplete recovery in cytosolic and microsomal incubation, the emergence of other metabolites was monitored. A desacryl-metabolite (m/z: 387.2 > 84.1, and 387.2 > 304.1) emerged, which was formed most likely due to esterase activity. Further metabolism of ICYS through *N*-acetyltransferase was performed by incubation of ICYS with a human liver S9 fraction pool fortified with acetyl-CoA for 1 h at 37 °C and pH 7.4. Through incubation of 500 nM ICYS with human liver cytosol in the presence of 1 mM Acetyl-CoA it was shown that ICYS can be converted to its mercapturic acid-conjugate (m/z: 604.1 > 473.1, and 604.1 > 304.1) at 6.27 ± 0.23 nM/min.

The involvement of ciPTEC GSTs was confirmed by using cell lysate as an enzyme source [20]. The formation of IGSH was enhanced by the presence of lysate and was inhibited by pre-incubating the ciPTEC lysate with 100 μM ethacrynic acid for 2.5 h, confirming the involvement of renal GSTs.

### ɣ-glutamyl transferase activity and inhibition

Degradation of GSH conjugates depends on the activity of ɣ-GT activity, an enzyme that is highly abundant in all tissues, especially liver, and that converts IGSH rapidly to ICGS. In vitro, IGSH showed a half-life of 0.5 h in human plasma. ɣ-GT from equine kidney was used to determine the approximate kinetic parameters of the conversion from IGSH to ICGS. The K_m_ was determined at 588 nM, and the V_max_ at 25.8 μmol∙min^−1^∙U^−1^. The human plasma and complete medium used contained 16 and 0.5 U/mL ɣ-GT, respectively.

**Fig. 5:**
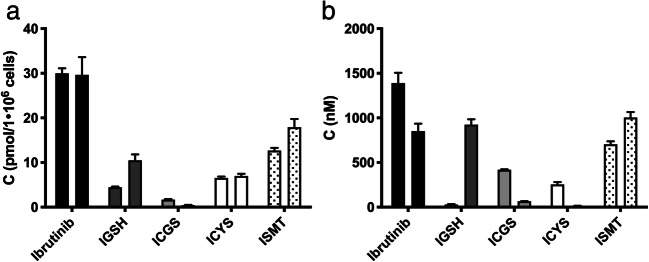
Metabolism of ibrutinib (2 μM) in ciPTEC after 4-h with the drug. Control (left bar) and inhibition with ethacrynic acid (right bar) of ɣ-glutamyl transferase in ciPTEC cell lysate (**a**) and incubation medium (**b**)

Metabolism of IGSH in ciPTEC could be blocked by acivicin, both intra- and extracellularly, as shown by accumulation of IGSH in Fig. 5 showing inhibition results with ethacrynic acid. Intracellular ibrutinib and ICYS concentrations were unchanged. Extracellularly, IGSH did accumulate, and no significant ICGS and ICYS formation was observed.

### Transport and cytotoxicity of ibrutinib and its metabolites in ciPTEC

**Fig. 6:**
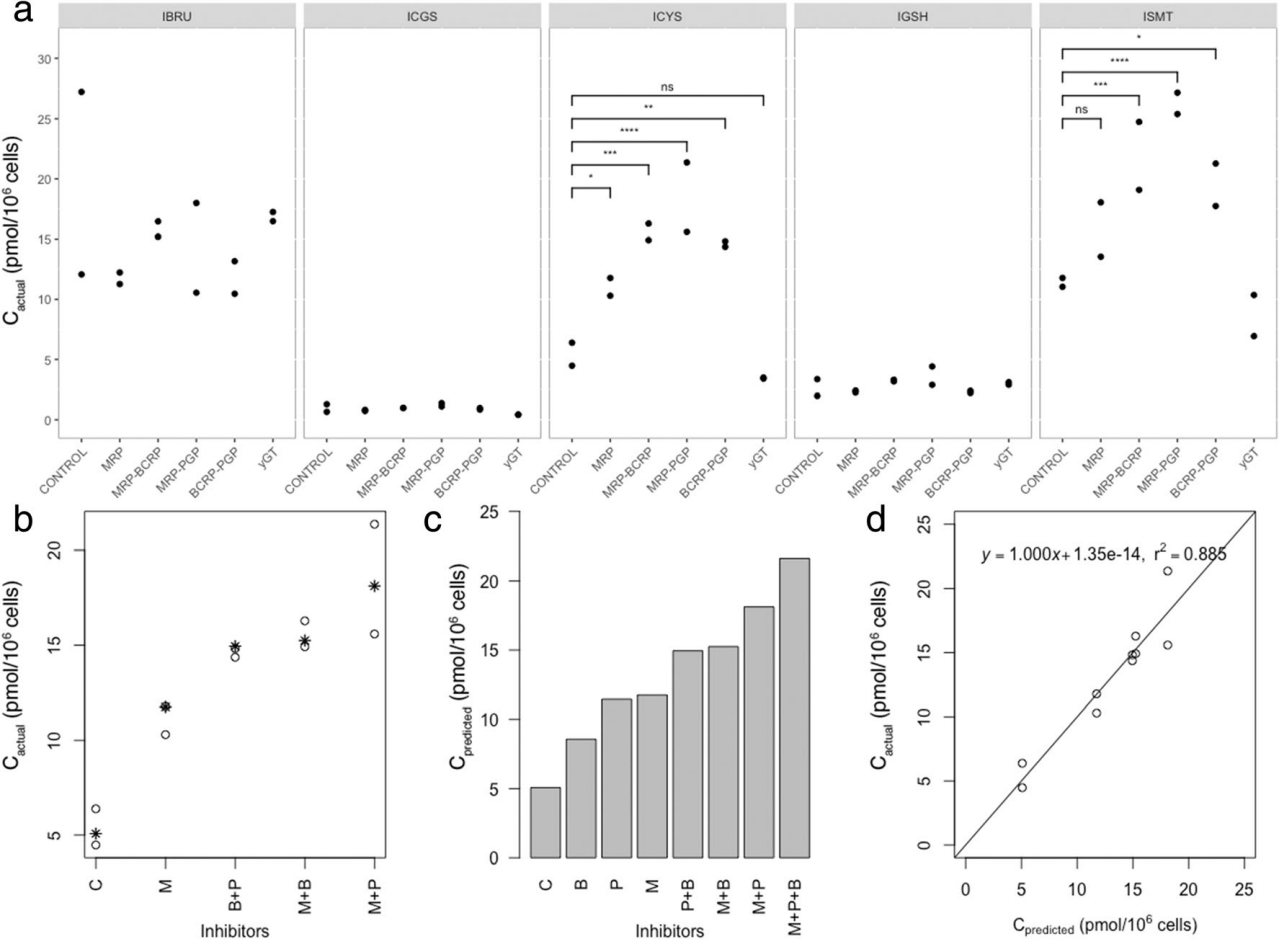
**a**) Intracellular concentrations of ibrutinib and all ISMTs in ciPTEC cells after 4-h incubation. Cells were co-incubated with different combinations of BCRP, P-gp, and MRP inhibitors. For ICYS, all combinations showed significant effects versus control (*p* < 0.05, *n* = 2, 4 h). **b**-**d**) Mixed effects linear regression model for the effect of the inhibitors (B: BCRP, M: MRP, P: PGP). **b**) The actual concentrations of the inhibitors (o) and in overlay the predicted values (*) (n = 2, 4 h). **c**) Predicted ICYS concentration in ciPTEC lysate for each inhibitor-combination. **d**) Visual analysis of regression analysis of predicted vs. actual values

**Fig. 7:**
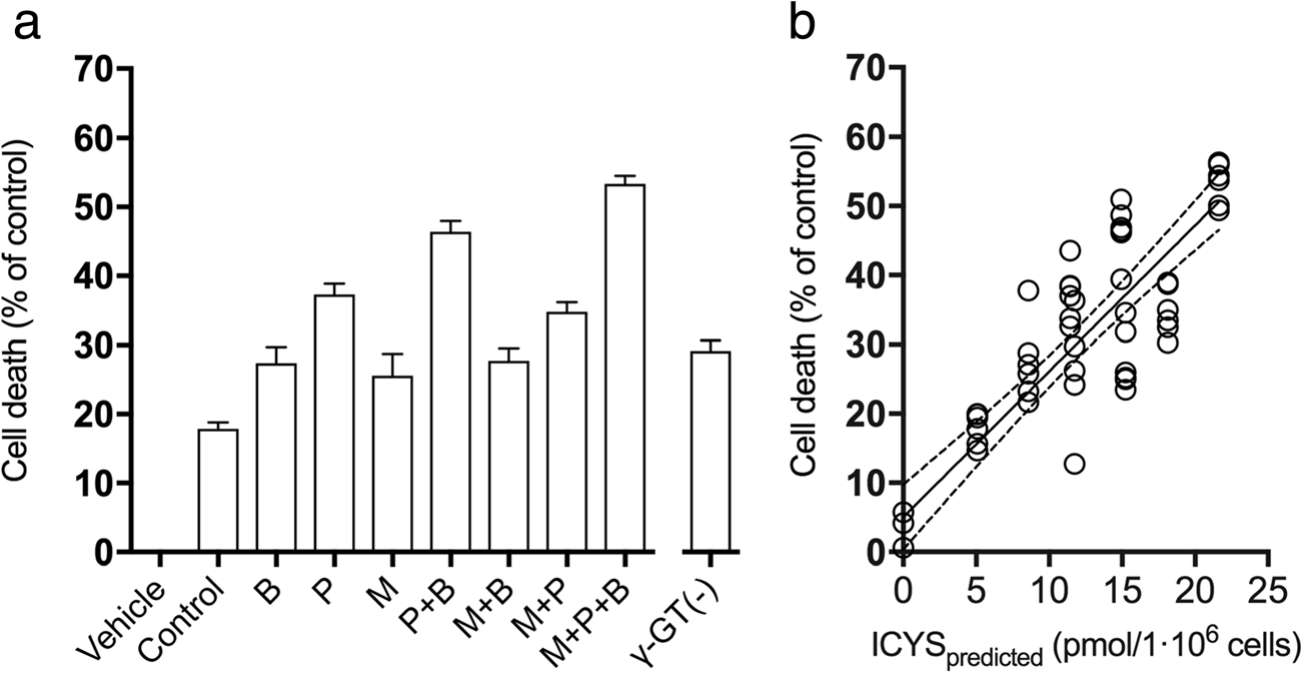
**a**) Toxicity of ibrutinib in presence of P-gp (P), BCRP (B) and MRP (M) inhibitors, single-inhibitor or any combination. All conditions showed a statistically significant increase in toxicity (mean ± SD, *n* = 6, 48 h). All conditions showed a significant effect versus control (2 μM ibrutinib, *P* values ≤0.05). **b**) Linear regression model of the predicted ICYS-concentration vs. cell viability shows a decrease in viability with rising ICYS concentrations (Y = −2.108·X + 94.97). The lines represent regression model ±95% confidence intervals

To investigate the importance of renal drug transporters for the pharmacokinetics of ibrutinib, ciPTEC were incubated with 2 μM ibrutinib in the presence and absence of inhibitors (5 μM) of the BCRP, P-gp and MRP2/4, namely Ko143, PSC833, and MK571, blocking the efflux of ibrutinib and its metabolites. The intracellular concentrations of ibrutinib and the ISMT metabolites were analyzed after 4 h of incubation (Fig. 6a). The isolated effects of each inhibitor and their combinations were extrapolated through a mixed effects linear regression model (Fig. 6c). MRPs inhibition resulted in the highest accumulation of ICYS, followed by the P-gp inhibition in ciPTEC. Combined inhibition of MRP and P-gp showed the strongest intracellular accumulation of ICYS. Of note, intracellular concentration of ibrutinib did not change significantly upon inhibition of the transporters. Inhibition of the efflux transporters P-gp and MRP2/4 resulted in higher levels of ibrutinib*-S-*glucuronide (*p* < 0.001), indicating a role of the efflux pumps in metabolite excretion. We further investigated whether increased accumulation of ICYS led to increased cytotoxicity. Single or combination inhibition of efflux transporters resulted in an increased cell death in ciPTEC (Fig. 7a), which correlated with the predicted ICYS concentration (Fig. 7b).

### Ibrutinib and ISMT-metabolites in mice

**Fig. 8:**
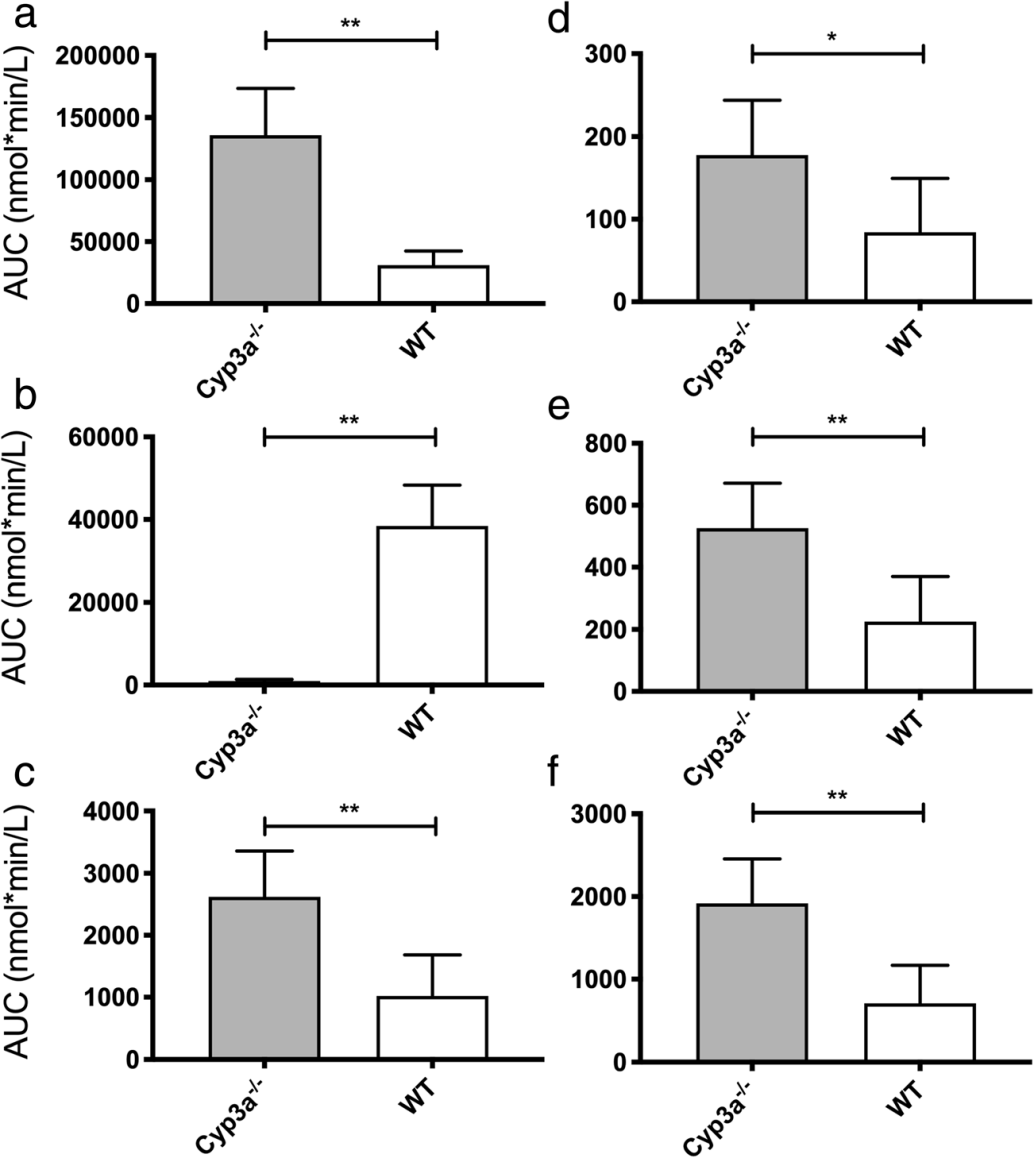
Cyp3a^−/−^ mice show increased extrahepatic metabolism of ibrutinib: AUC_0–20 min_ of **a**) ibrutinib, **b**) DHI, **c**) sum of IGSH, ICGS and ICYS, **d**) IGSH, **e**) ICGS, **f**) ICYS (*: *P* ≤ 0.05, **: *P* ≤ 0.01)

**Fig. 9:**
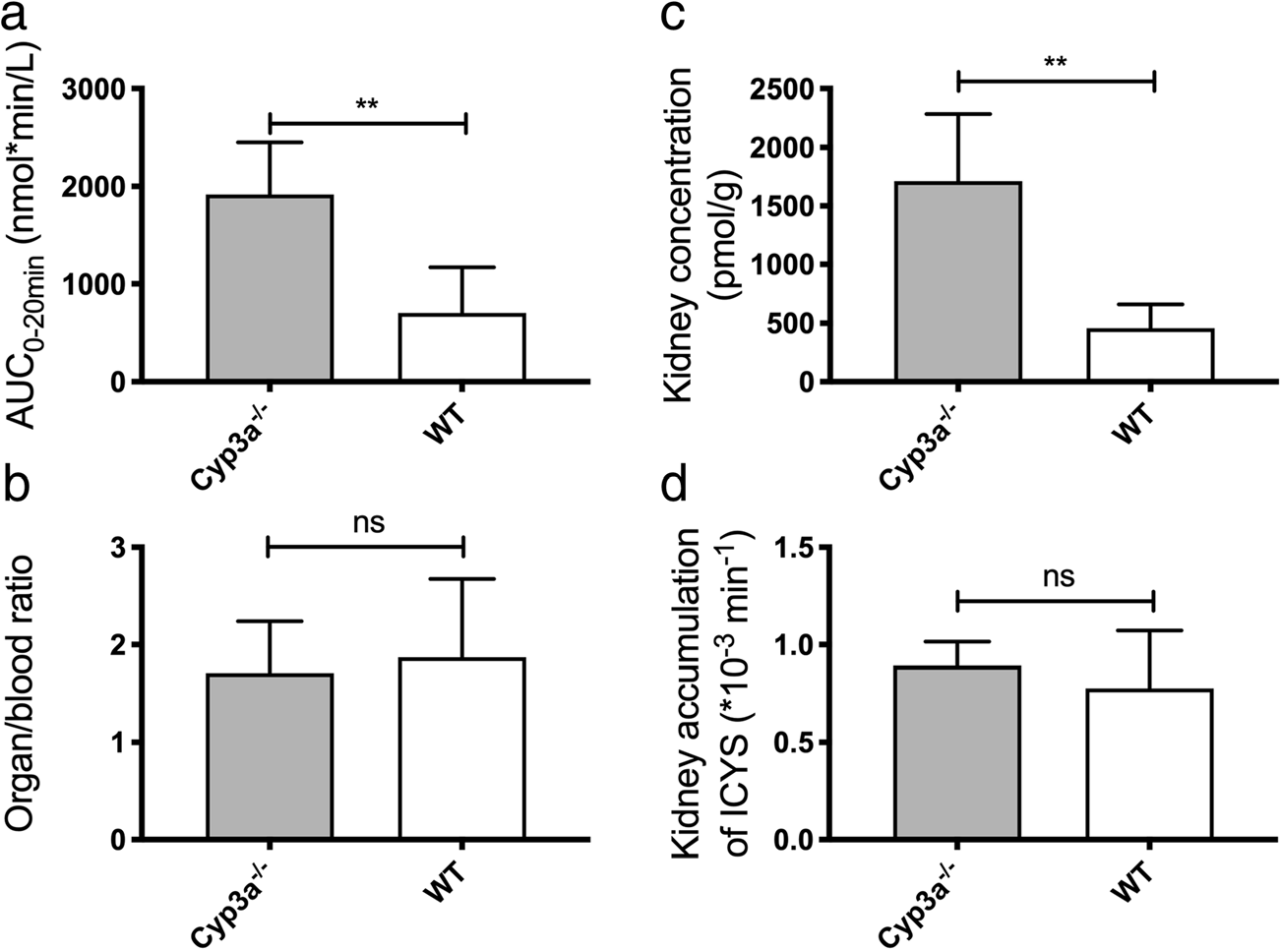
Cyp3a^−/−^ mice show increased ICYS concentrations: **a**) AUC_0–20 min_ for ICYS, **b**) Kidney/blood ratio for ICYS, **c**) Absolute ICYS concentration in kidney, **d**) ICYS kidney accumulation (ns: *P* > 0.05, **: P ≤ 0.01)

To evaluate the role of CYP3A in ibrutinib metabolism, a PK study was performed in Cyp3a knockout mice. Cyp3a^−/−^ (FVB/NRj genetic background) mice showed an increase of 2.5-fold (AUC_0–20 min_ 2620 ± 740 vs 1018 ± 663 nmol·min/L for WT) for the sum of all SMT-metabolites (2.0 ± 0.4% of the ibrutinib exposure (Fig. 8). The SMT-conjugate exposure is mainly due to the cysteine metabolite, to which IGSH and ICGS were rapidly converted in vivo. In spleen, no IGSH was found, and only moderate amounts of ICGS and ICYS were present. High IGSH concentrations were present in liver homogenates, and ICYS seems to accumulate in kidneys, as expected. In Cyp3a-deficient mice, the ISMT accounted for a major part of the quantified metabolites (72.7 ± 11.8% vs 2.4 ± 1.1% for FVB/NRj), and renal concentrations increased to 2.6-fold of the FVB/NRj-mice (Fig. 9).

## Discussion

**Fig. 10:**
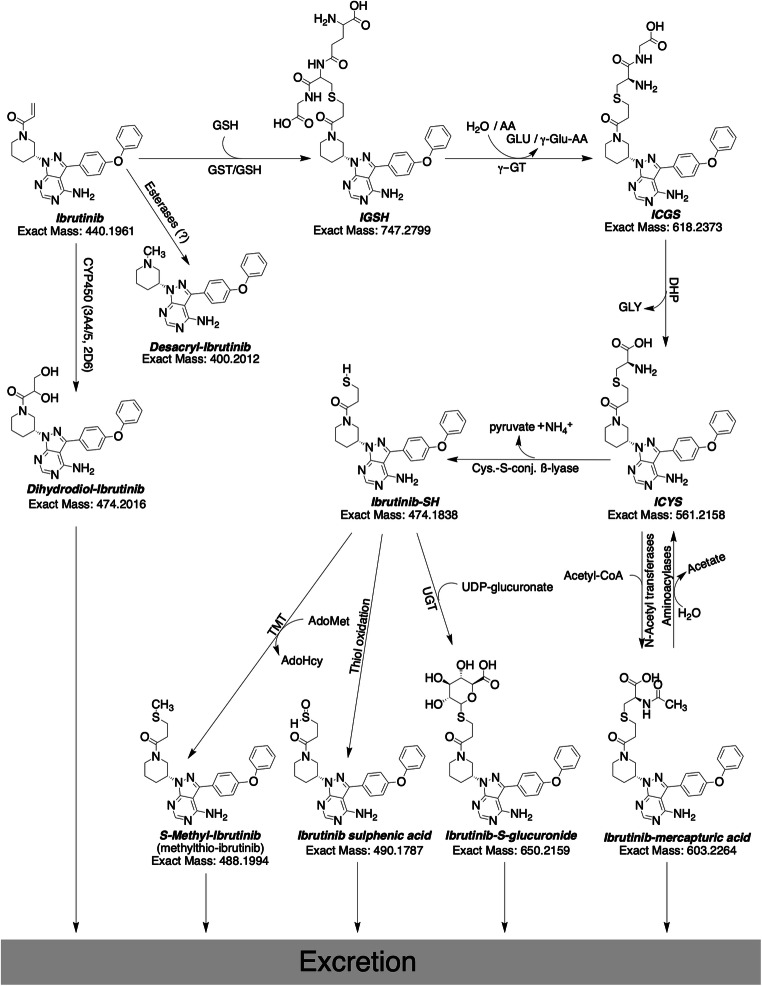
Proposed ibrutinib metabolic pathways for the extrahepatic metabolism through the glutathione cycle and further bioactivation and biotransformation. Exclamation marks denote reactive groups (electrophiles). AA: Amino acid, Acetyl-CoA: Acetyl-Coenzyme A, AdoHcy: S-adenosyl-homocysteine, AdoMet: S-adenosyl-methionine, DHP: Dehydropeptidase I, ɣ-GT: ɣ-glutamyl transferase, GLU: Glutamine, GLY: Glycine, GSH: Glutathione (H-GLU-CYS-GLY-OH), GST: Glutathione-S-Transferase, TMT: Thiol S-methyltransferase, UDP: uridine diphosphate, UGT: UDP-glucuronosyltransferase

To gain a complete picture of ibrutinib metabolism, research into the extrahepatic metabolism of ibrutinib was started. It revealed the extent to which GSH-conjugation can play a role and was especially focused on human. We evaluated that for humans it might be up to 30% of the ibrutinib related material. For mice, however, the relative contribution of the ISMT-metabolites was low compared to humans. For wild-type mice, roughly 2% was metabolized through this route, which might be due to the short residence time of ibrutinib in mice. With a plasma half-live of half an hour in the WT mice, compared to 4 to 12 h for humans, ibrutinib has less time to react to thiols [31]. However, it was clear that the lack of Cyp3a resulted in a substantial increase of plasma and kidney ICYS levels. We hypothesized that formation of cysteine-metabolites by renal dipeptidases could accelerate the bioactivation pathway through cysteine-*S*-conjugate ß-lyase. This would cleave the cysteine residue, resulting in a thiol group on the pharmacophore [18]. The electrophilic thiol of ibrutinib-SH is then able to form the unwanted protein conjugates [32], and is inactivated by either *S*-conjugate reductases to form a sulphenic acid metabolite, thiomethyl-transferases to form the *S*-methylated metabolite, or glucuronide-transferases to form the *S*-glucuronide [33]. A graphical representation of GSH-mediated metabolism of ibrutinib, along with the bioactivation pathway is schematically shown in Fig. 10. Mammalian cysteine-*S*-conjugate ß-lyases are mainly amino-acid metabolizing enzymes that have ß-lyase activity causing an unwanted side reaction. While most of the previous work on the enzymes mainly focused on bioactivation of halogenated alkenes, more recent metabolic studies have revealed that ß-lyase activity is more important in the metabolism of chemotherapeutic agents [34]. ß-lyase is an enzyme expressed in renal tubules that is often involved in bioactivation and nephrotoxicity [35].

The bioactivation of ICYS to thio-ibrutinib (ibrutinib-SH) was indirectly proven by the formation of ibrutinib-*S*-glucuronide, and possibly very minor amounts of *S*-methyl-ibrutinib. Due to direct intracellular formation of ICYS, the formation of this metabolite could not be prevented by blocking ɣ-GT. The cell-viability could be well predicted by the incubation with a small subset of inhibitors with a main role of MRP-inhibition by MK571. With the use of inhibitors, the intracellular concentrations of ibrutinib, IGSH and ICGS remained mainly unchanged, whereas the ICYS concentrations differed significantly between the inhibitor concentrations. This indicates that the toxicity was most likely due to ICYS accumulation, as supported by the correlation between intracellular ICYS and cell viability. Ibrutinib is metabolized through GSTs in both liver and kidney, as well as non-enzymatic metabolism by direct conjugation with GSH. One study quantified the formation of IGSH in in vitro samples [9], but not in more complex systems like living cells, or even whole organisms. IGSH was previously not detected, but other SMT-metabolites, ICGS and mercapturic acid conjugates were [6]. To gain more quantitative insight in the extrahepatic metabolism, we investigated both in vitro and in vivo SMT-metabolite formation of ibrutinib. The decrease in IGSH formation in HLMs could be explained by the degradation of GSH by ɣ-GT present in the microsomes. In ciPTEC, the complete metabolism to eventually ICYS became visible and quantifiable. In cells, GSH is continuously supplied through regeneration of GSSG, or de-novo synthesis [8]. CiPTEC are capable of metabolizing ibrutinib at a relatively high rate. The kinetics of ibrutinib in medium showed an apparent distribution phase, after which the compounds were metabolized at 87 pmol/h/1·10^6^ cells. The ICYS metabolites appeared to be formed intracellularly in the renal cells as a result of direct conjugation. Ɣ-GT is only located extracellularly [36], and ICGS and ICYS did not appear to be transported back into the cells, most likely due to the absence of the organic anion transporters (OAT1 and OAT3) in the parent ciPTEC used in this study [37]. This was demonstrated by direct incubations of ciPTEC with IGSH, and was supported by co-incubation with acivicin, a potent ɣ-GT inhibitor, along with a high reactivity towards molecular cysteine. This metabolic ‘escape’ route makes inhibiting ɣ-GT meaningless in preventing ICYS-related toxicity, unlike for instance for cisplatin [32], although the latter study was not a human trial.

GST-mediated metabolism could act as a resistance mechanism in cancer cells through upregulation of GSTs [38, 39]. An example of this is busulfan GST-mediated resistance [27]. α,β-Unsaturated aldehydes are known substrates of GST-π [40]. It is known that cells in CLL are capable of upregulating GSTs, even as a resistance mechanism. Analysis of CLL lymphocyte GST activity showed a 2-fold increase in cells from chlorambucil-resistant patients over those from untreated patients and healthy individuals [41]. MCL cells can also show abnormal GST activity [42].

The metabolism of ibrutinib through the glutathione cycle might not play a major role under normal conditions [6], but this route becomes a formidable alternative pathway when the oxidative metabolism is impaired. So, in case of enzyme inhibition by concomitant medication, or genetic defects in the involved enzymes, extrahepatic metabolism starts to play a more prominent role [10]. This was shown by the vast difference in metabolite concentrations between the 24 patient (A and B) samples, and the samples from patient C receiving voriconazole. Voriconazole is a substrate and inhibitor of CYP2C19, CYP2C9, CYP3A4, but appears to not have a significant effect on a number of drug transporters. However, it did show some inhibition of BCRP [43]. For this patient, the slow elimination is supposed to be caused by uptake of an intestinal reservoir as opposed to entero-hepatic circulation [44]. The lack of AUCs for ibrutinib and the metabolites in the uninhibited state makes comparing the inhibition case with the normal population troublesome. While we did not find any circulating ibrutinib-SH, or the metabolites that originate from it, this was also not expected. Ibrutinib-SH is most likely only a very minor metabolite which is converted quickly into ibrutinib-*S*-glucuronide and is then excreted in the urine. Nonetheless, renal toxicity was shown in vitro with up to 50% cell death. MRP inhibition led to the greatest accumulation of ICYS, followed closely by P-gp. It is logical that MRP inhibition has the largest effects because of the substrate specificity of the transporter with respect to drug-conjugates [45]. Concomitant therapy that might interact with transporters could contribute to toxicity. With the sum of ISMTs corresponding to roughly 30% of the ibrutinib-related material it was shown that ibrutinib metabolism through the thio-metabolism with glutathione plays a prominent role, especially when CYP3A4 is inhibited. Overall, more in-depth knowledge on ibrutinib biotransformation routes was obtained, creating more insight into the pharmacokinetics of ibrutinib and its main metabolites. The knowledge gathered is useful to guide PK-study design and a led to better understanding of drug-drug interactions, as well as providing a mechanistic explanation for ibrutinib-mediated nephrotoxicity.

## Electronic supplementary material

ESM 1 (PDF 624 kb)
