# Supplementary material for: Extrahepatic metabolism of ibrutinib
Source: Invest New Drugs. 2020 Jul 4;39(1):1–14. doi: 10.1007/s10637-020-00970-x (PMC7851014; doi:10.1007/s10637-020-00970-x)
Supplement: Supplementary file 1 — (PDF 624 kb) [file 10637_2020_970_MOESM1_ESM.pdf]

Extrahepatic metabolism of ibrutinib

Johannes J.M. Rood, Amer Jamalpoor, Stephanie van Hoppe, Matthijs J. van Haren, Roeland E. Wasmann, Manoe J. Janssen, Alfred H. Schinkel, Rosalinde Masereeuw, Jos H. Beijnen, Rolf W. Sparidans

Supplemental Tables

Supplemental Table 1: Patient data for 24 samples of 2 patients (A and B) for ibrutinib, DHI, IGSH, ICGS, and ICYS.

Index	Patient	Sample ID	T (h)	Dose	Group	Analyte	Concentration (nM)
1	A	5	0.5	1xdd 420mg	abs	ibru	11.8
2	A	3	0.8	1xdd 560mg	abs	ibru	3.5
3	A	21	0.8	1xdd 420mg	abs	ibru	143.2
4	A	6	0.8	1xdd 420mg	abs	ibru	14.6
5	A	18	1.2	1xdd 420mg	abs	ibru	117.5
6	A	2	1.4	1xdd 420mg	abs	ibru	6.6
7	A	16	1.5	1xdd 420mg	abs	ibru	11.6
8	B	12	2.4	1xdd 560mg	max	ibru	551.2
9	A	13	2.5	1xdd 420mg	max	ibru	683.1
10	B	4	2.8	1xdd 560mg	max	ibru	234.3
11	B	1	3.3	1xdd 560mg	max	ibru	309.2
12	A	14	3.8	1xdd 420mg	max	ibru	151.2
13	B	20	3.8	1xdd 560mg	max	ibru	195.7
14	B	17	3.9	1xdd 560mg	max	ibru	578.0
15	B	23	4.1	1xdd 560mg	max	ibru	497.1
16	B	7	4.1	1xdd 560mg	max	ibru	359.7
17	B	24	4.4	1xdd 560mg	max	ibru	323.2
18	B	9	4.4	1xdd 560mg	max	ibru	192.3
19	A	10	22.2	1xdd 420mg	trough	ibru	7.0
20	A	11	22.6	1xdd 420mg	trough	ibru	4.3
21	A	8	22.7	1xdd 420mg	trough	ibru	8.9
22	A	19	23.3	1xdd 420mg	trough	ibru	5.5
23	A	15	NA	1xdd 420mg	[a]	ibru	15.8
24	A	22	NA	1xdd 420mg	[a]	ibru	0.9
25	A	5	0.5	1xdd 420mg	abs	dhi	51.6
26	A	3	0.8	1xdd 560mg	abs	dhi	17.5
27	A	21	0.8	1xdd 420mg	abs	dhi	188.2
28	A	6	0.8	1xdd 420mg	abs	dhi	48.3
29	A	18	1.2	1xdd 420mg	abs	dhi	137.1
30	A	2	1.4	1xdd 420mg	abs	dhi	43.9
31	A	16	1.5	1xdd 420mg	abs	dhi	24.0
32	B	12	2.4	1xdd 560mg	max	dhi	449.9
33	A	13	2.5	1xdd 420mg	max	dhi	403.7
34	B	4	2.8	1xdd 560mg	max	dhi	345.6
35	B	1	3.3	1xdd 560mg	max	dhi	419.9
36	A	14	3.8	1xdd 420mg	max	dhi	195.3
37	B	20	3.8	1xdd 560mg	max	dhi	524.1
38	B	17	3.9	1xdd 560mg	max	dhi	542.0
39	B	23	4.1	1xdd 560mg	max	dhi	468.0

40	B	7	4.1	1xdd 560mg	max	dhi	523.3
41	B	24	4.4	1xdd 560mg	max	dhi	484.1
42	B	9	4.4	1xdd 560mg	max	dhi	393.3
43	A	10	22.2	1xdd 420mg	trough	dhi	90.7
44	A	11	22.6	1xdd 420mg	trough	dhi	45.3
45	A	8	22.7	1xdd 420mg	trough	dhi	86.6
46	A	19	23.3	1xdd 420mg	trough	dhi	30.5
47	A	15	NA	1xdd 420mg	[a]	dhi	59.3
48	A	22	NA	1xdd 420mg	[a]	dhi	<LLOQ
49	A	5	0.5	1xdd 420mg	abs	igsh	<LLOQ
50	A	3	0.8	1xdd 560mg	abs	igsh	0.5
51	A	21	0.8	1xdd 420mg	abs	igsh	<LLOQ
52	A	6	0.8	1xdd 420mg	abs	igsh	<LLOQ
53	A	18	1.2	1xdd 420mg	abs	igsh	<LLOQ
54	A	2	1.4	1xdd 420mg	abs	igsh	<LLOQ
55	A	16	1.5	1xdd 420mg	abs	igsh	<LLOQ
56	B	12	2.4	1xdd 560mg	max	igsh	0.8
57	A	13	2.5	1xdd 420mg	max	igsh	1.5
58	B	4	2.8	1xdd 560mg	max	igsh	3.5
59	B	1	3.3	1xdd 560mg	max	igsh	2.6
60	A	14	3.8	1xdd 420mg	max	igsh	0.5
61	B	20	3.8	1xdd 560mg	max	igsh	0.8
62	B	17	3.9	1xdd 560mg	max	igsh	1.9
63	B	23	4.1	1xdd 560mg	max	igsh	2.2
64	B	7	4.1	1xdd 560mg	max	igsh	1.5
65	B	24	4.4	1xdd 560mg	max	igsh	1.4
66	B	9	4.4	1xdd 560mg	max	igsh	0.8
67	A	10	22.2	1xdd 420mg	trough	igsh	<LLOQ
68	A	11	22.6	1xdd 420mg	trough	igsh	<LLOQ
69	A	8	22.7	1xdd 420mg	trough	igsh	<LLOQ
70	A	19	23.3	1xdd 420mg	trough	igsh	<LLOQ
71	A	15	NA	1xdd 420mg	[a]	igsh	<LLOQ
72	A	22	NA	1xdd 420mg	[a]	igsh	<LLOQ
73	A	5	0.5	1xdd 420mg	abs	icgs	5.4
74	A	3	0.8	1xdd 560mg	abs	icgs	9.7
75	A	21	0.8	1xdd 420mg	abs	icgs	11.9
76	A	6	0.8	1xdd 420mg	abs	icgs	7.3
77	A	18	1.2	1xdd 420mg	abs	icgs	11.1
78	A	2	1.4	1xdd 420mg	abs	icgs	6.7
79	A	16	1.5	1xdd 420mg	abs	icgs	2.2
80	B	12	2.4	1xdd 560mg	max	icgs	73.2
81	A	13	2.5	1xdd 420mg	max	icgs	412.5

82	B	4	2.8	1xdd 560mg	max	icgs	159.9
83	B	1	3.3	1xdd 560mg	max	icgs	97.1
84	A	14	3.8	1xdd 420mg	max	icgs	72.5
85	B	20	3.8	1xdd 560mg	max	icgs	78.0
86	B	17	3.9	1xdd 560mg	max	icgs	226.1
87	B	23	4.1	1xdd 560mg	max	icgs	238.6
88	B	7	4.1	1xdd 560mg	max	icgs	190.9
89	B	24	4.4	1xdd 560mg	max	icgs	160.2
90	B	9	4.4	1xdd 560mg	max	icgs	88.1
91	A	10	22.2	1xdd 420mg	trough	icgs	3.7
92	A	11	22.6	1xdd 420mg	trough	icgs	3.0
93	A	8	22.7	1xdd 420mg	trough	icgs	5.6
94	A	19	23.3	1xdd 420mg	trough	icgs	0.6
95	A	15	NA	1xdd 420mg	[a]	icgs	5.1
96	A	22	NA	1xdd 420mg	[a]	icgs	<LLOQ
97	A	5	0.5	1xdd 420mg	abs	icys	5.1
98	A	3	0.8	1xdd 560mg	abs	icys	3.9
99	A	21	0.8	1xdd 420mg	abs	icys	12
100	A	6	0.8	1xdd 420mg	abs	icys	5.6
101	A	18	1.2	1xdd 420mg	abs	icys	9.9
102	A	2	1.4	1xdd 420mg	abs	icys	5.0
103	A	16	1.5	1xdd 420mg	abs	icys	2.3
104	B	12	2.4	1xdd 560mg	max	icys	209.5
105	A	13	2.5	1xdd 420mg	max	icys	185.8
106	B	4	2.8	1xdd 560mg	max	icys	92.8
107	B	1	3.3	1xdd 560mg	max	icys	77.1
108	A	14	3.8	1xdd 420mg	max	icys	32.1
109	B	20	3.8	1xdd 560mg	max	icys	78.7
110	B	17	3.9	1xdd 560mg	max	icys	143.4
111	B	23	4.1	1xdd 560mg	max	icys	194.3
112	B	7	4.1	1xdd 560mg	max	icys	170.3
113	B	24	4.4	1xdd 560mg	max	icys	210.5
114	B	9	4.4	1xdd 560mg	max	icys	89.2
115	A	10	22.2	1xdd 420mg	trough	icys	5.0
116	A	11	22.6	1xdd 420mg	trough	icys	4.7
117	A	8	22.7	1xdd 420mg	trough	icys	6.4
118	A	19	23.3	1xdd 420mg	trough	icys	2.6
119	A	15	NA	1xdd 420mg	[a]	icys	12.1
120	A	22	NA	1xdd 420mg	[a]	icys	< LLOQ

[a] omitted (no group)

Supplemental Table 2: Concomitant drugs for the 73 yr. old patient receiving a lowered dose due to co-administration of a potent CYP3A4-inhibitor.

Drug	DD	Dose	Route	Inhibits	Metabolism
Clemastine	2	1 mg	IV	CYP2D6	-
Cyanocobalamin	1	6 mg	n.g.	-	-
Enteral feeding	continuous	-	n.g.	-	-
Furosemide	continuous	2 mg/h	IV	-	-
Glucose 5%	continuous	42 ml/h	IV	-	-
Haloperidol	1	1 mg	IV	CYP2D6	**CYP3A4**
Ibrutinib	1	140 mg	n.g.	-	**CYP3A4**, 2D6
Macrogol	2	2 sachets	n.g.	-	-
Magnesium sulphate	1	15 mg	n.g.	-	-
Metoclopramide	3	10 mg	IV	-	*CYP2D6*
Midazolam	continuous	10 mg/h	IV	-	**CYP3A4**
Monosodium phosphate	continuous	2 mmol/h	IV	-	-
Nadroparin	1	2850 IE	SC	-	-
Parenteral nutrition	continuous	84 ml	IV	-	-
Propofol	continuous	100 mg/h	IV	-	-
Salbutamol/ipratropium 1/0,2mg/ml	1-2	2.5 ml	IH	-	-
Sufentanil	continuous	10 µg/h	IV	-	*CYP3A4*
Tobramycin/colistin/ amphotericin B oral paste	4	1 g	Oral	-	-
Tobramycin/colistin/ amphotericin B oral suspension	4	10 mL	n.g.	-	-
Voriconazole	2	340 mg	IV (1h)	CYP3A4, CYP2C9, CYP2C19	**CYP3A4, CYP2C9, CYP2C19**

DD: number of daily doses, IH: inhaled (after nebulizing), IV: intravenous, n.g.: nasogastric tube, SC: sub-cutaneous, **BOLD**: relevant interaction by strong inhibitors of that enzyme*, *italicized:* deemed not-relevant* Underlined: relevant for ibrutinib. (* Relevance as evaluated by the Royal Dutch Pharmacists Association (kennisbank.knmp.nl, visited 17-06-2018)).
